# Predicting the Uniaxial Compressive Strength of Cement Paste: A Theoretical and Experimental Study

**DOI:** 10.3390/ma18153565

**Published:** 2025-07-30

**Authors:** Chunming Lian, Xiong Zhang, Lu Han, Weijun Wen, Lifang Han, Lizhen Wang

**Affiliations:** 1 Key Laboratory of Advanced Civil Engineering Materials of Education Ministry, School of Material Science and Technology, Tongji University, 4800 Cao’an Road, Shanghai 201804, China; 2 China Construction Eighth Bureau Science and Technology Construction Co., Ltd., 899 Gaoke West Road, Shanghai 201804, China; whosname12@163.com (L.H.); wenweijun@cscec.com (W.W.); hanlifang0810@126.com (L.H.); lizhen_w@163.com (L.W.)

**Keywords:** cement paste, compressive strength, mineral admixtures, density correction, strength prediction model

## Abstract

This study presents a progressive strength prediction model for cement paste based on the hypothesis that compressive strength is governed by the microstructural compactness of hydration products. A three-stage modeling framework was developed: (1) a semi-empirical model for pure cement paste incorporating water-to-cement ratio and paste density; (2) a density-corrected effective water–cement ratio ${\left(w/c\right)}_{eff}$ that accounts for the physical effects of mineral additives including fly ash, slag, and limestone powder; and (3) a hydration-informed strength model incorporating curing age and temperature through an equivalent hydration degree $\alpha \left(t_{e}\right)$. Experimental validation using over 60 cement paste mixes demonstrated high predictive accuracy, with coefficients of determination up to 0.97. The proposed model unifies the influence of binder composition, packing density, and curing conditions into a physically interpretable and practically applicable formulation. It enables early-age strength prediction of blended cementitious systems using only routine mix and density parameters, supporting performance-based mix design and optimization. The methodology provides a robust foundation for extending compactness-based modeling to more complex cementitious materials and structural applications.

## 1. Introduction

### 1.1. Background and Significance of Cement Paste Strength

Concrete, the backbone of modern infrastructure, derives its mechanical strength from hardened cement paste, which serves as the binding matrix for aggregates [1]. While the compressive strength of concrete is a key design parameter, it is intrinsically governed by the strength characteristics of the cement paste itself. Accurate prediction of cement paste strength is, thus, fundamental for optimizing mix design, ensuring long-term structural reliability, and meeting the performance requirements of diverse construction applications [2,3]. For instance, in the United States, ASTM C109/C109M [4] outlines, providing a benchmark for the strength contribution of the paste phase [5]. Similarly, European standards such as EN 196-1 [6] specify methods for determining the compressive strength of cement, with typical 28-day strengths for common Portland cement often ranging from 32.5 MPa to 52.5 MPa depending on the cement class [7]. In China, GB/T 17671 [8] sets comparable standards, emphasizing the importance of minimum strength criteria for quality control and structural integrity across various applications [9].

Traditional concrete design approaches often rely on empirical testing, such as the 28-day compressive strength standard. While widely used, these methods introduce delays in construction workflows and offer limited adaptability to new material systems [10]. Predictive models that estimate cement paste strength from early-age properties or mix parameters would enhance design efficiency and support performance-based engineering.

This research is based on the hypothesis that the uniaxial compressive strength of cement paste is directly proportional to the microstructural density of its hydration products. This concept offers a physically meaningful and mechanistically grounded foundation for developing strength prediction models based on observable composition and curing variables.

### 1.2. Review of Existing Strength Models for Cementitious Materials

Over the past century, strength models for cementitious materials have evolved from empirical formulas to more mechanistic frameworks. Abrams [11] established a classic inverse relationship between compressive strength and water–cement (w/c) ratio, while Powers [12] proposed the gel–space ratio theory, introducing a physical interpretation based on hydration product formation and porosity reduction. Feret similarly developed a porosity-based formulation [13]. These early models successfully described broad trends but lacked microstructural resolution.

Later research introduced additional influencing factors such as aggregate–paste interface properties [14,15,16], paste volume optimization [15], and time-dependent hydration–drying interactions [17]. The work of de Larrard [18], which models concrete as a binary composite of aggregates and cement paste, emphasized the load-bearing role of the paste phase and paved the way for linking macroscopic strength to paste behavior.

However, the widespread use of modern mineral additions—particularly fine powders introduced either intentionally or as by-products—has introduced new complexities. While much attention has been paid to supplementary cementitious materials (SCMs) such as fly ash and ground granulated blast-furnace slag (GGBFS) [19,20,21,22], comparatively less focus has been placed on fine powders unintentionally introduced via aggregate systems, such as limestone or granite powder from crushed aggregates. These aggregate-derived fines, often present in significant amounts in manufactured sand or recycled aggregates, can influence hydration kinetics, pore structure refinement, and, ultimately, the microstructural densification of the cement paste [23,24].

Traditional w/c-based models do not adequately capture the effects of these materials, whose roles are not limited to dilution but also include nucleation effects, filler compaction, and potential chemical interactions, depending on their mineralogy. Thus, there is a growing need for predictive models that consider the influence of aggregate-originated mineral fines alongside standard binder parameters.

Meanwhile, microstructure-based models, such as those proposed by Maekawa et al. [25] and Bentz and Garboczi [26], offer deeper insight into the hydration process and pore structure evolution. Jennings [27] highlighted the role of densified C–S–H as a critical strength contributor, supported by nanoindentation and thermogravimetric analyses [28,29]. However, these models are often computationally intensive and require inputs that are impractical for routine engineering use.

Moreover, although studies by Thomas [30], Papadakis [31], and others have explored pozzolanic reactivity and filler effects, the modeling of aggregate-derived fines remains fragmented. Environmental and curing conditions—such as temperature, relative humidity, and sealing conditions—further affect hydration and strength gain but are often oversimplified in existing models [32].

### 1.3. Research Objectives and Scope

This research aims to address the lack of a comprehensive, mechanistically grounded, and experimentally validated model for predicting cement paste compressive strength across a range of mix compositions and curing regimes, with particular attention to the role of aggregate-derived fine powders.

This study is structured around three main objectives:

1. To develop a theoretical and experimental model for the uniaxial compressive strength of pure cement paste, grounded in the hypothesis that strength is directly proportional to the volumetric density of hydration products. While the fundamental concept of strength–density correlation has roots in classical theories [33,34], our novelty lies in developing a pragmatic yet physically meaningful model that integrates readily measurable parameters (such as paste density) with a refined mechanistic understanding to provide highly accurate predictions across diverse cementitious systems, particularly pure cement pastes. The water–cement ratio will serve as the primary input variable in the initial formulation.
2. To extend the model to include the effects of mineral admixtures, with a specific focus on fine powders introduced via aggregate systems (e.g., limestone powder, granite powder). These materials will be treated as functional components that influence hydration kinetics, filler packing, and the resulting microstructure.
3. To incorporate curing conditions and age into the model, capturing the effects of environmental variables such as temperature, humidity, and curing duration. These parameters are essential to accurately represent hydration progression and microstructural densification over time.

By integrating theoretical modeling with experimental validation, this study seeks to produce a practical and robust strength prediction framework that accounts for real-world material variability and construction conditions.

## 2. Theoretical Framework for Strength Prediction of Pure Cement Paste

### 2.1. Fundamental Hypothesis: Strength–Microstructural Compactness Relationship

The uniaxial compressive strength of hardened cement paste is fundamentally governed by the intricate internal structure formed through cement hydration. This study is built upon the core hypothesis that the compressive strength of hardened cement paste is directly proportional to the microstructural compactness of its hydration products. This assumption is consistent with foundational principles in materials science and composite mechanics, which assert that a material’s ability to resist mechanical stress improves with the continuity, density, and homogeneity of its internal solid phase [35,36]. In cement-based materials, such compactness arises from the progressive accumulation and efficient arrangement of hydration products within the available pore system.

Furthermore, the concept of microstructural compactness is intimately linked with the setting behavior of cement paste. Setting time, typically measured as initial and final set, marks the transition of the cement paste from a plastic, workable state to a rigid, solidified mass. This transition is driven by the initial formation and interlocking of hydration products, primarily C-S-H, which begin to bridge the cement particles and fill the interstitial spaces. Consequently, a faster setting time often indicates an accelerated initial hydration rate and a more rapid development of early-age microstructure and, thus, strength [37]. While setting time itself is not a direct measure of ultimate strength, it serves as an important indicator of the early-stage densification and stiffening processes that lay the foundation for subsequent strength gain. A well-controlled setting process is critical to ensure proper consolidation and prevent issues like segregation or excessive bleeding, which can compromise the final microstructural compactness and, by extension, the strength of the hardened paste.

Cement hydration generates a set of solid-phase products—primarily calcium silicate hydrate (C–S–H), calcium hydroxide (CH), and ettringite (AFt)—which gradually occupy the originally water-filled spaces among cement grains. As the hydration process proceeds, the growing solid volume reduces pore connectivity and creates a denser and more robust matrix. The resulting improvements in strength can be attributed to three interrelated microstructural effects:

- Reduced Porosity: Lower total and connected pore volumes diminish stress concentration sites, consequently increasing the effective load-bearing solid area.
- Improved Solid-Phase Continuity: A more interconnected C–S–H network facilitates more efficient internal stress transfer throughout the material under applied loads.
- Suppressed Defect Formation: Denser microstructures inherently inhibit the formation and propagation of critical flaws, such as large pores or microcracks, which often trigger premature failure [27].

Thus, microstructural compactness serves as a physically interpretable and quantifiable proxy for the paste’s intrinsic capacity to sustain compressive stress.

### 2.2. Model for Pure Cement Paste Strength

Building upon the principle that compressive strength correlates with the volumetric fraction of solid phases in hardened cement paste, Féret’s early work [34] proposed a widely cited empirical model, as shown in Equation (1):

(1) $$f_{cp}=K{\left(\frac{v_{c}}{v_{c}+v_{w}+v_{a}}\right)}^{n}$$

where $f_{c}$ is the compressive strength of the paste, $v_{c}$ is the volume of cement, $v_{w}$ is the volume of mixing water, $v_{a}$ is the volume of entrapped air, K is a constant, and n is an exponent. This model suggests that paste strength is directly proportional to the volumetric ratio of cement within the paste.

Extending this principle, F. Larrard [18] introduced a model that incorporated cement type and 28-day standard mortar strength:

(2) $$f_{cp}=A\times {Rc}_{28}{\left(\frac{v_{c}}{v_{c}+v_{w}+v_{a}}\right)}^{n}$$

where ${Rc}_{28}$ is the 28-day compressive strength of standard cement mortar, while A and n are dimensionless shape correction coefficients.

Although both formulations support the strength–solid content relationship, their direct application to hardened pastes is hindered by the difficulty in measuring entrapped air volume ($v_{a}$) post hardening. To improve the practicality of the model, the volumetric ratio in Equation (1) can be transformed using mass and density relations:

(3) $$\frac{v_{c}}{v_{c}+v_{w}+v_{a}}=\frac{m_{c}/\rho_{c}}{m_{p}/\rho_{p}}=\frac{\rho_{p}}{\rho_{c}\left(1+w/c\right)}$$

where $m_{c}$ is the mass of cement, $\rho_{c}$ and $\rho_{p}$ are the densities of cement and hardened paste, respectively, and w/c is the water-to-cement ratio. This transformation enables strength to be expressed as a function of directly measurable quantities.

Nevertheless, the true determinant of hardened paste strength is the volume fraction of solid hydration products and unreacted cement, rather than the original binder volume alone. While direct quantification of hydration product volume remains complex, the bulk density $\rho_{p}$ of the hardened paste serves as an effective proxy for microstructural compactness. Therefore, a strength prediction model based on measurable quantities such as $\rho_{p}$, w/c, and α (degree of hydration) offers a more practical and physically meaningful approach to model cement paste strength.

### 2.3. Extension for Mineral Admixtures

The incorporation of mineral additives such as fly ash (FA), ground granulated blast-furnace slag (GGBFS), and fine limestone powder (originating from aggregate fines) alters the strength development of cement paste. At early ages (e.g., 28 days), experimental evidence and previous research indicate that the effect of these materials is primarily physical—through pore refinement, particle packing, and dilution—while their chemical reactivity contributes minimally to hydration product volume [38,39,40].

In accordance with the core hypothesis of this study—that compressive strength is proportional to the compactness of the hardened microstructure—the influence of mineral additives can be accounted for via their impact on the volumetric density of the paste. Rather than modeling the complex secondary reactions of FA and GGBFS, which are slow and require extensive characterization, we adopt a practical density-based correction to the water–cement ratio.

To this end, we define an equivalent or effective water–cement ratio ${\left(w/c\right)}_{eff}$, which integrates the volumetric influence of mineral additives and entrained air through a density ratio. The formulation is as follows:

(4) $${\left(w/c\right)}_{eff}=w/c\times {\left(\frac{\rho_{cp}}{\rho_{p}}\right)}^{\gamma }$$

where w and c denote the masses of water and cement, respectively, $\rho_{cp}$ is the theoretical density of a paste composed solely of water and cement, and $\rho_{p}$ is the measured density of the paste, which accounts for the inclusion of mineral additives (FA, GGBFS, limestone powder) as well as entrapped air. γ is an empirical coefficient reflecting the nonlinear sensitivity of strength to compactness variation.

This correction accounts for the following:

- Dilution effects due to partial cement replacement by less reactive materials;
- Filler and packing effects through improved particle dispersion and reduced effective porosity;
- Entrapped air, which lowers actual density and weakens microstructural continuity.

The effective water–cement ratio ${\left(w/c\right)}_{eff}$, therefore, provides a measurable and physically meaningful parameter that captures the net impact of additives on microstructural compactness.

Incorporating Equation (4) into the base strength formulation leads to the following expression for the compressive strength of blended cement paste.

(5) $$f_{cp}=A\alpha \left(t_{e}\right)R_{c_{28}}{\left[\frac{\rho_{p}}{\rho_{c}\left(1+{\left(w/c\right)}_{eff}\right)}\right]}^{n}$$

Here, $\alpha \left(t_{e}\right)$ represents the degree of hydration of cement and A is a strength coefficient related to the efficiency of hydration products in forming a load-bearing network. As secondary reactions of FA and GGBFS are minor within the modeling timeframe, $\alpha \left(t_{e}\right)$ is defined only in terms of cement hydration.

This formulation allows the model to remain compactness-driven, experimentally verifiable, and extensible to various blended binder systems without overcomplicating the predictive framework. It aligns closely with the theoretical foundation while improving applicability for practical mix design and early-age strength assessment. Unlike many computationally intensive microstructure models [25,41] or purely empirical approaches, the novelty of our model lies in its ability to offer a robust and practical framework that unifies the diverse effects of various mineral additives—including often-overlooked aggregate-derived fines—on early-age paste strength through a physically interpretable and density-corrected water-to-cement ratio, making it readily applicable for routine engineering use.

## 3. Materials and Experimental Methods

### 3.1. Cementitious Materials

The cement used in this study was Conch brand Ordinary Portland Cement (P·O 42.5), complying with the Chinese standard GB 175 [42]. This cement is manufactured by Anhui Conch Cement Company Limited, a major cement producer headquartered in Wuhu, Anhui, China. The physical properties of the cement are summarized in Table 1, including its specific surface area, which was determined by the Blaine air permeability method according to GB/T 1345 [43].

The mineral additives included Class II fly ash (FA) conforming to GB/T 1596 [44], S95-grade ground granulated blast-furnace slag (GGBFS) as per GB/T 18046 [45], and limestone powder (LP) as per GB/T 35164 [46]. All three of these materials were supplied by Beijing Zhengyuan Yiqing New Material Technology Co., Ltd. (Beijing, China). The density of each powder was measured using a Le Chatelier flask and kerosene according to Chinese standard GB/T 208 [47]. Particle size distributions were measured using a BT-9300LD laser diffraction particle size analyzer, from Bettersize Instruments Ltd. located in Dandong, China. Their specific surface areas were also determined by the Blaine air permeability method as per GB/T 1345. Results are listed in Table 2.

The chemical and typical mineralogical compositions of these supplementary cementitious materials (SCMs) are provided in Table 3.

**Figure 1:**
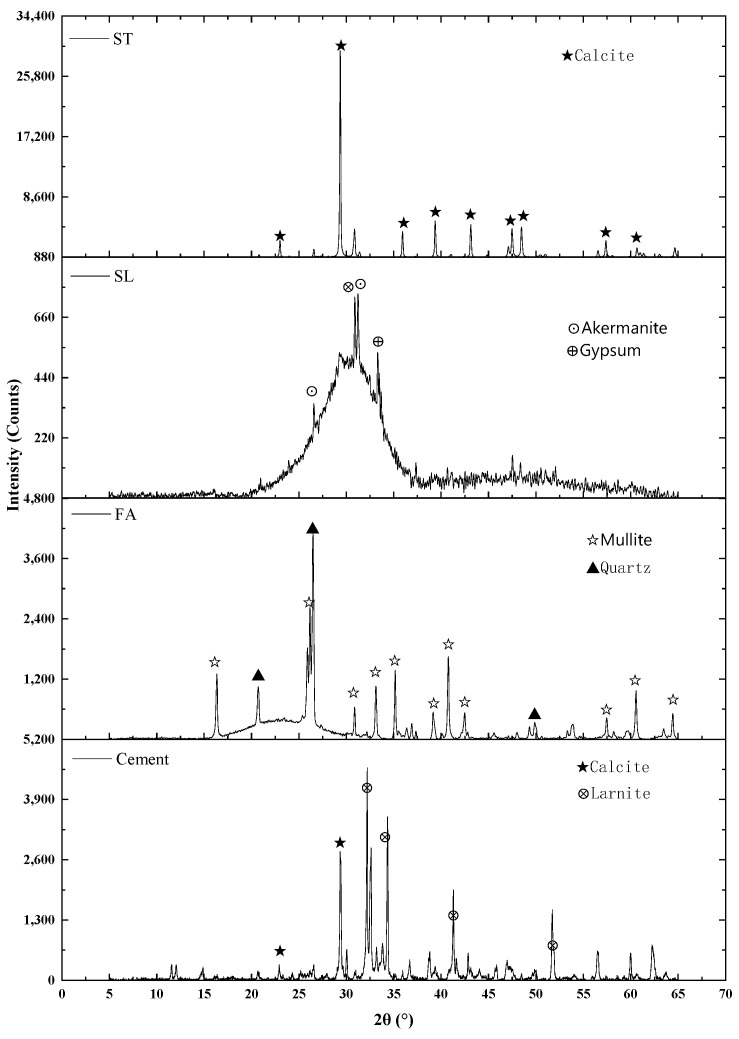
X-ray diffractogram of cement and mineral admixtures.

Figure 1 displays the X-ray diffractograms of the cement and various mineral admixtures, revealing their respective crystalline phases.

### 3.2. Mix Proportions and Specimen Preparation

To investigate the factors influencing the strength of hardened cement paste, five water-to-cement ratios (w/c) were selected for pure cement paste. Pastes were cast in 40 mm × 40 mm × 40 mm molds, cured under standard conditions (20 ± 1 °C and RH ≥ 95%) for 28 days, and tested for uniaxial compressive strength. Mixed details and strength results are presented in Table 4.

Experimental hydration degree models were introduced for later integration with the strength model. The hydration degree $\alpha \left(t_{e}\right)$ was defined following Schindler [48], who proposed a temperature- and age-dependent expression:

(6) $$\alpha \left(t_{e}\right)=\alpha_{max}\cdot exp\left[-{\left(\frac{\tau }{t_{e}}\right)}^{\beta }\right]$$

with hydration parameters τ=36 h, β=0.6 for P·O 42.5 cement and $\alpha_{max}$ as the ultimate hydration degree, predicted using the Mills equation [49].

(7) $$\alpha_{max}=\frac{1.031\cdot w/c}{0.194+w/c}$$

The equivalent age $t_{e}$ can be calculated based on the Arrhenius law:

(8) $$t_{e}=\int_{0}^{t}exp\left[\frac{E_{a}}{R}\left(\frac{1}{293}-\frac{1}{T+273}\right)\right]dt$$

where $E_{a}$ is Activation Energy, R is the universal gas constant hydration model, and *T* is temperature. Equations (6)–(8) were used to represent strength evolution under different curing regimes. Strength development at multiple ages under standard curing for various w/c ratios is shown in Table 5.

### 3.3. Experimental Setup and Visual Documentation

**Figure 2:**
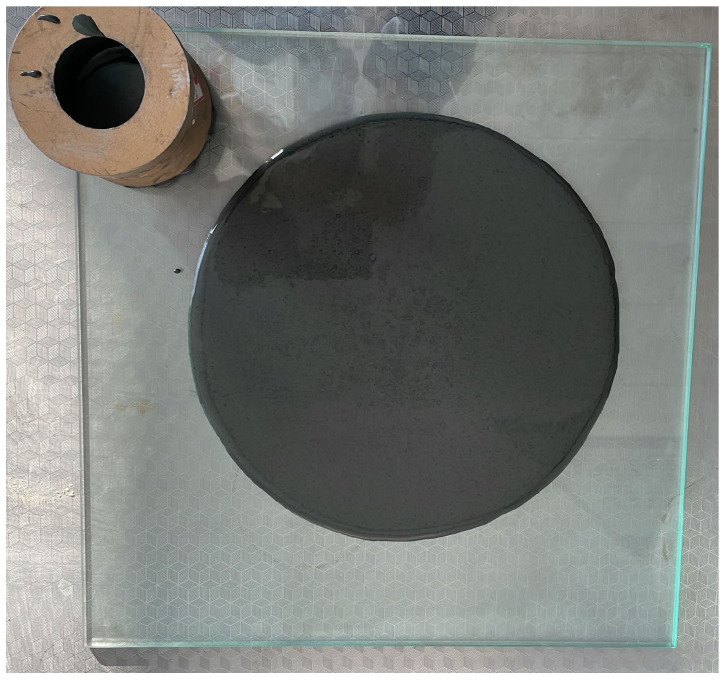
Photographs of cement paste specimens.

**Figure 3:**
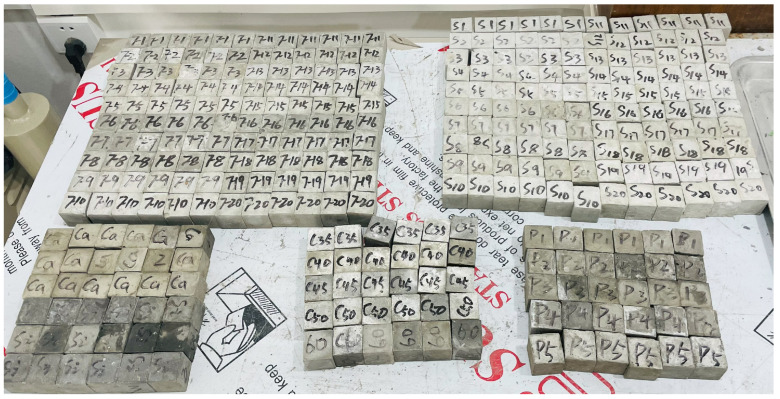
Uniaxial compressive strength specimens.

To enhance the clarity and reproducibility of the experimental procedures, key aspects of the sample preparation are visually documented. Figure 2 presents photographs of the cement paste flowability testing, illustrating the fluid characteristics of the paste. Figure 3 shows the cement paste cubic specimens after demolding, displaying the prepared test samples.

### 3.4. Cement Pastes with Mineral Additives

To evaluate the effect of mineral additives on paste strength, fly ash (FA), slag (GGBFS), and limestone powder (LP) were used at various replacement levels. A total of 65 mix designs were tested, varying in water-to-binder ratio (w/b), additive type, and dosage. All specimens were prepared and cured under the same conditions as the pure cement pastes. A complete set of mixed proportions and 28-day compressive strength results is presented in Table 6.

This comprehensive dataset supports the model development and calibration described in Section 2, particularly the density-based correction of the water–cement ratio used in strength prediction.

## 4. Results and Analysis

### 4.1. Strength of Pure Cement Paste

**Figure 4:**
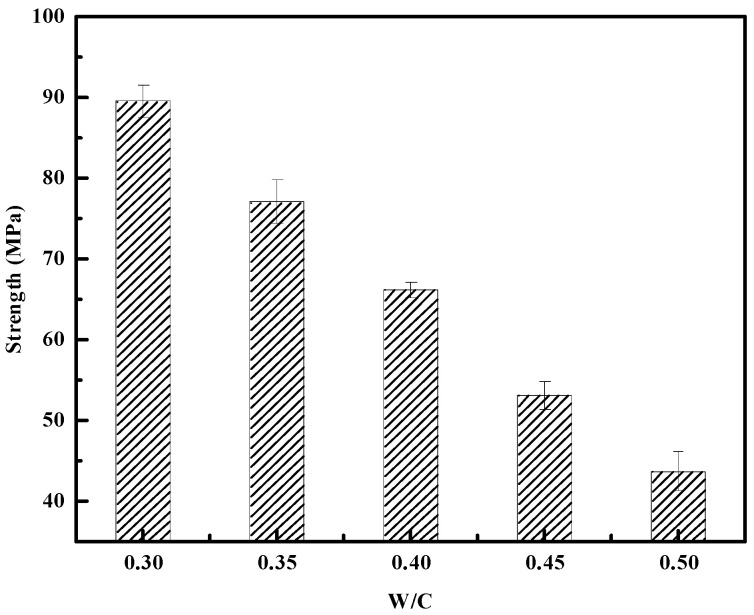
Relationship between w/c ratio and 28-day compressive strength of pure cement paste.

Figure 4 presents the relationship between compressive strength and water-to-cement ratio (w/c) for the five pure cement paste mixes listed in Table 3. As expected, compressive strength decreases with an increasing w/c ratio. The highest 28-day strength of 89.5 MPa was observed at w/c = 0.30, while the lowest was 43.6 MPa at w/c = 0.50. This trend confirms that lower water content results in denser microstructures and higher hydration product concentration consistent with the strength–compactness hypothesis.

**Figure 5:**
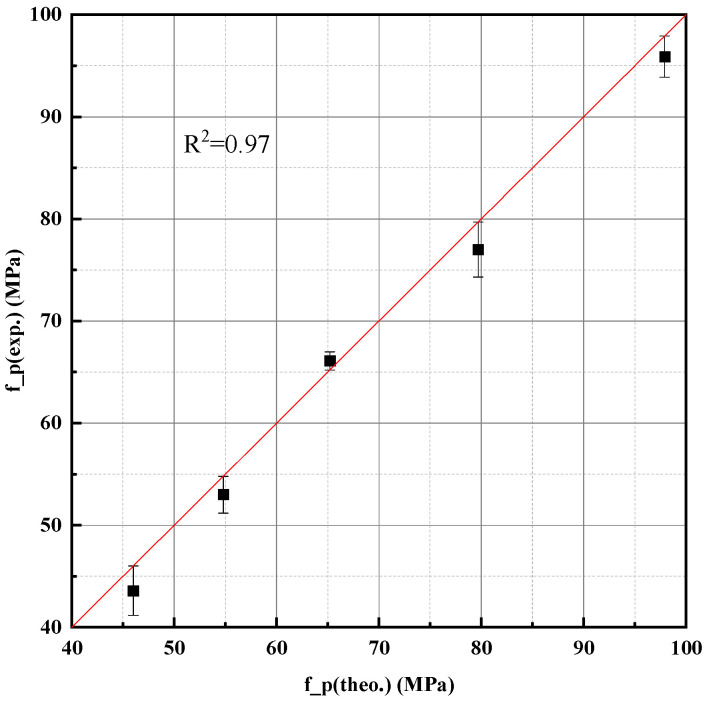
Model fitting results for pure cement paste based on Equation (5).

A refined strength prediction model for pure cement paste was established based on the theoretical density $\rho_{p}$ and the measured water-to-cement ratio. The model takes the following form:

(9) $$f_{cp}=13.37R_{c_{28}}{\left(\frac{\rho_{p}}{\rho_{c}\left(1+w/c\right)}\right)}^{2.77}$$

where $f_{cp}$ is compressive strength of hardened paste (MPa); $R_{c_{28}}$ is 28-day mortar strength of cement, measured as 43.2 MPa; $\rho_{c}$ is density of cement (2990 kg/m^3^); $\rho_{p}$ is measured density of hardened paste; w/c is water-to-cement ratio; and 13.37 and 2.77 are empirical shape parameters. This model is semi-empirical in nature, built on a physical understanding of pore volume and strength correlation, while incorporating regression analysis for parameter calibration. The coefficient of determination (R^2^) for the fit was 0.97, indicating high predictive accuracy (Figure 5).

Compared to fully theoretical or purely empirical models, Equation (5) integrates structural insight with experimental data, offering both physical interpretability and practical reliability. It effectively quantifies the influence of w/c ratio on strength and provides a microstructural rationale for strength formation in hardened cement paste.

In addition to 28-day strength values, the compressive strength development of pure cement pastes at different w/c ratios under standard curing was measured at multiple ages (3, 7, 14, 28, and 90 days).

The strength data demonstrates clear time-dependent development and strong sensitivity to the water-to-cement ratio. Samples with lower w/c ratios not only showed higher initial strength, but also maintained greater long-term strength increments.

To interpret this behavior, the degree of hydration was calculated using the equivalent age hydration model described in Section 2. The model was calibrated against this multi-age dataset. A modified strength prediction equation was proposed.

(10) $$f_{cp}=34.7\alpha \left(t_{e}\right)R_{c_{28}}{\left[\frac{\rho_{p}}{\rho_{c}\left(1+w/c\right)}\right]}^{3.1}$$

**Figure 6:**
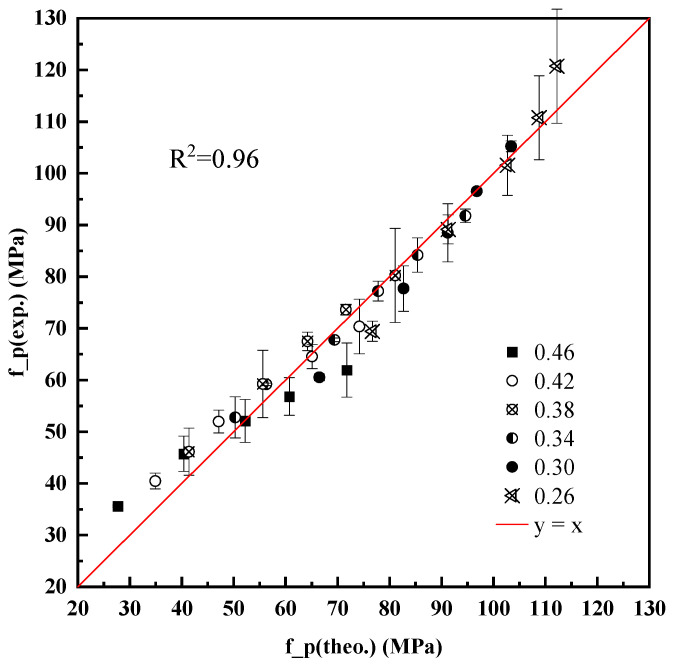
Predictive accuracy of the density-corrected strength model using Equation (10).

The model demonstrates high accuracy in describing the time-dependent compressive strength development of pure cement pastes under standard curing conditions. Considering degrees of hydration, the compressive strength prediction model shown in Equation (10) offers high precision, with the determination coefficient R^2^ = 0.96 (Figure 6). This confirms that the model can reliably quantify strength development based on the combined influence of hydration progression and structural compactness.

Furthermore, since the hydration degree can be adjusted according to curing conditions, Equation (10) can also be extended to predict the compressive strength of cement paste under different curing regimes and at various curing ages. This reinforces the model’s general applicability and adaptability for practical engineering prediction tasks.

It is also critical to acknowledge that, beyond hardened properties, the fresh properties of cement paste, particularly its flowability and the feasibility of its transportation and placement, are paramount for practical construction applications. While not directly quantified as output parameters in our strength prediction model, all mix designs investigated in this study were meticulously prepared to ensure adequate workability for proper casting and consolidation into molds. Mixes with lower w/c ratios, while yielding higher ultimate strength, inherently present challenges in flowability and require careful mixing procedures to achieve a homogeneous paste that can be effectively handled and transported without segregation, highlighting the practical trade-offs involved in mix design optimization.

### 4.2. Influence of Mineral Additives on Strength

To evaluate the impact of mineral additives on strength development, the experimental data from Table 5 were first plotted using the water-to-binder ratio (w/b) as the horizontal axis. As shown in Figure 4, the 28-day compressive strength generally decreases with an increasing w/b ratio. However, the degree of sensitivity and the slope of the strength reduction curves vary significantly across different types and dosages of mineral additives.

**Figure 7:**
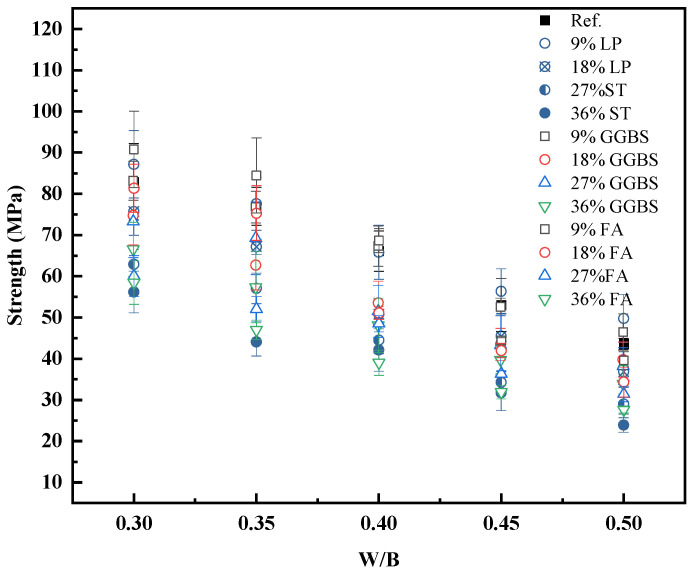
Influence of w/b ratio on 28-day compressive strength of cement pastes with mineral additives.

The trends reveal that the inclusion of mineral additives leads to distinct strength behaviors depending on their physical and chemical characteristics (Figure 7). Because of differences in early-age reactivity, fly ash, slag, and limestone powder modify not only the total binder content, but also the packing and hydration environment of the paste. As a result, strength–w/b relationships are non-uniform and cannot be described using a single equation for all compositions.

**Figure 8:**
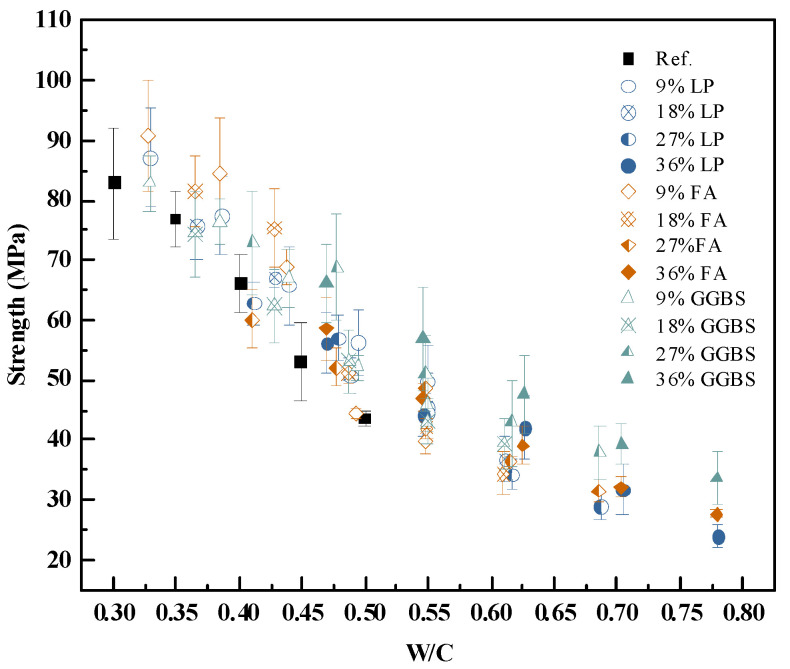
Influence of w/c ratio on 28-day compressive strength of cement pastes with mineral additives.

The scatter and inconsistency in Figure 4 suggest that using w/b as the independent variable leads to considerable variability in the results due to the additive type and content. In contrast, when the water-to-cement ratio (w/c) is used instead, the strength trends become more cohesive. Figure 8 presents the 28-day compressive strength plotted against w/c for all tested mixtures.

Compared to Figure 7, the dispersion in Figure 8 is significantly reduced, and the overall trend—decreasing strength with increasing w/c—is more clearly observed across all binder systems. This suggests that w/c is a more dominant factor governing early-age paste strength than w/b, particularly when mineral additives act primarily through physical mechanisms.

The strength behavior supports the hypothesis that mineral additives at early ages primarily influence strength through volume displacement. In this sense, they replace an equivalent volume of water in the paste system and act as micro-aggregates that are inert after hardening.

Based on this understanding, the corrected water-to-cement ratio is defined in Equation (4). The exponent was calibrated by minimizing the error between predicted and measured strength values across all samples. The best-fit value was found to be n = 1.92.

Substituting Equation (4) into the generalized strength model results in the following.

(11) $$f_{cp}=34.7\alpha \left(t_{e}\right)R_{c_{28}}{\left[\frac{\rho_{p}}{\rho_{c}\left(1+{w/c}^{\prime }\right)}\right]}^{3.1}$$

**Figure 9:**
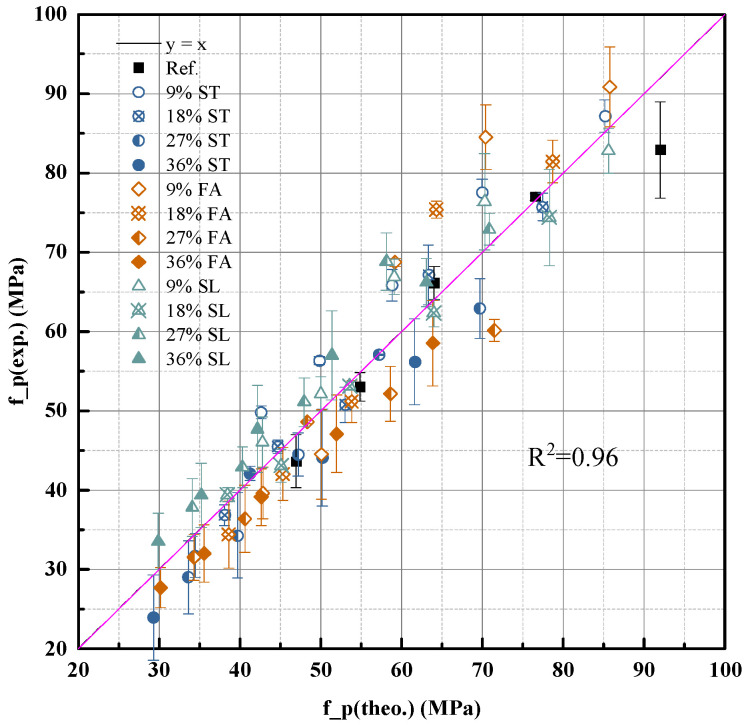
Predictive accuracy of the density-corrected strength model using Equation (11).

The predictive performance of this revised model is shown in Figure 9 with a coefficient of determination, demonstrating high consistency between predicted and experimental values.

This result further confirms that using a density-based correction to the water–cement ratio can effectively unify the effects of different mineral additives on strength. The model provides a compactness-driven framework with sufficient accuracy and generality for early-age paste strength prediction in blended systems.

## 5. Conclusions and Outlook

This study established a progressive strength prediction model for cement paste based on the core hypothesis that compressive strength is governed by the microstructural compactness of hydration products. Through a three-stage modeling framework and extensive experimental validation, the following key conclusions were drawn:

- For pure cement pastes, compressive strength is highly correlated with the water-to-cement ratio (w/c). A semi-empirical model based on paste density and theoretical packing (Equation (5)) was proposed and verified, with a determination coefficient R^2^ = 0.97.
- Strength development over time was modeled using the degree of hydration calculated via an equivalent age approach. This hydration-informed model (Equation (10)) demonstrated excellent agreement with experimental data over multiple curing ages (from 3 to 90 days), with R^2^ = 0.96.
- Mineral additives (fly ash, slag, limestone powder) affect strength primarily through physical filler and dilution effects at early ages. Their influence can be accurately captured by correcting the w/c ratio using measured paste density.
- A density-corrected water–cement ratio was introduced and validated, enabling a unified expression (Equation (11)) that predicts the strength of blended cement pastes with high accuracy using only measurable mix parameters and hydration degree.
- The final model accommodates variation in curing temperature and time, enabling strength prediction across curing conditions by adjusting hydration degree.
- The primary novelty of this research lies in developing a pragmatic yet physically grounded strength prediction model that unifies the effects of diverse mineral additives, including aggregate-derived fines, through a density-corrected water-to-cement ratio. This approach offers a significant advancement over traditional empirical models and computationally intensive microstructural simulations by providing high predictive accuracy with readily measurable parameters, thus enhancing applicability for practical mix design and early-age strength assessment.

This strength prediction model holds significant engineering value. It provides a physically interpretable and experimentally grounded framework that can be practically implemented using routinely measurable parameters. By linking strength to hydration degree and paste density, the model enables the performance-based design of cement pastes incorporating mineral additives such as fly ash, slag, and limestone powder. This supports more sustainable cementitious systems by allowing for the precise evaluation and optimization of binder combinations in early-age design. This advanced understanding and predictive capability are particularly vital for the global construction industry, forming the bedrock for durable and sustainable infrastructure. The optimized design of cement paste is fundamental to a vast array of applications, ranging from high-performance structural elements in buildings and bridges to specialized geotechnical and repair solutions.

Looking forward, future work can focus on extending the model to later-age strength prediction by incorporating the time-dependent pozzolanic activity of mineral admixtures, especially fly ash and slag. Additional developments may include integrating this compactness-based framework with micromechanical simulations or finite element tools to link microstructure evolution with macro-scale mechanical performance. Further investigations can also evaluate the influence of chemical admixtures, shrinkage-compensating materials, and water-reducing agents, expanding the model’s applicability. Moreover, adapting the model to mortar and concrete systems by considering the role of the interfacial transition zone and aggregate packing could enhance its utility for practical structural applications.

Overall, the results of this study provide a robust foundation for the strength prediction of cement-based materials and offer technical insight into the design, evaluation, and optimization of blended cementitious systems under varied curing and material conditions.

## Figures and Tables

**Table 1:** Physical properties of P·O 42.5 cement.

Specific Surface Area (m^2^/kg)	Density (kg/m^3^)	Standard Consistency (%)	Initial Setting Time (min)		Final Setting Time (MPa)		Compressive Strength (MPa)	
317	2990	28.4	193	266	5.3 (3 d)	9.3 (28 d)	19.3 (3 d)	43.2 (28 d)

**Table 2:** Physical properties of cementitious powders.

Material	Density (g/cm^3^)	Specific Surface Area (m^2^/kg)	Surface Area per Volume (m^2^/m^3^)
Cement (C)	2.99	315.2	0.94
Fly Ash (FA)	2.35	453.4	1.07
Slag (GGBFS)	2.88	343.6	0.99
Limestone Powder (LP)	2.7	311.2	0.84

**Table 3:** Chemical and mineralogical compositions of cement and mineral additives.

Parameter	Unit	Cement	FA	GGBFS	LP
SiO_2_	%	19.5	36.5	36.0	2.0
CaO	%	63.5	25.0	43.0	54.0
Al_2_O_3_	%	5.0	17.5	15.0	0.5
MgO	%	1.5	6.0	6.0	0.5
Fe_2_O_3_	%	3.5	2.5	1.5	0.2
SO_3_	%	2.0	5.0	0.3	0.1
Na_z_O_eq_	%	0.5	0.6	0.6	0.1
LOI	%	0.6	0.7	0.5	43.0

**Table 4:** Pure cement paste mix proportions and 28-day strength.

Mix ID	Cement (g)	Water (g)	w/c Ratio	28 d Compressive Strength (MPa)
1	592	178	0.3	89.5
2	549	192	0.35	77
3	511	205	0.4	66.1
4	479	215	0.45	53
5	450	225	0.5	43.6

**Table 5:** Development of different water–cement ratio intensity under standard maintenance conditions.

Serial Number	w/c	Compressive Strength (MPa)				
		3 d	7 d	14 d	28 d	90 d
1	0.46	27.7	40.4	52.2	60.7	71.8
2	0.42	34.9	47.1	56.3	65.1	74.2
3	0.38	41.4	55.6	64.2	71.6	81.1
4	0.34	50.3	69.4	77.8	85.4	94.6
5	0.30	66.5	82.7	91.2	96.8	103.4
6	0.26	76.7	91.3	102.7	108.8	112.2

**Table 6:** Cement pastes with mineral additives: mix proportions and 28-day compressive strength.

Mix ID	Cement (g)	LP (g)	GGBFS (g)	FA (g)	Water (g)	w/b Ratio	28 d Compressive Strength (MPa)
1	450	0	0	0	225	0.5	44
2	592	0	0	0	178	0.3	86
3	549	0	0	0	192	0.35	77
4	511	0	0	0	205	0.4	66
5	479	0	0	0	215	0.45	53
6	532	53	0	0	175	0.3	87
7	493	49	0	0	190	0.35	72
8	460	46	0	0	202	0.4	61
9	431	43	0	0	213	0.45	50
10	406	40	0	0	223	0.5	42
11	473	104	0	0	173	0.3	76
12	439	96	0	0	188	0.35	67
13	410	90	0	0	200	0.4	51
14	384	84	0	0	211	0.45	46
15	362	79	0	0	221	0.5	37
16	416	154	0	0	171	0.3	66
17	387	143	0	0	185	0.35	57
18	361	134	0	0	198	0.4	45
19	339	125	0	0	209	0.45	34
20	319	118	0	0	219	0.5	29
21	360	203	0	0	169	0.3	56
22	335	188	0	0	183	0.35	44
23	313	176	0	0	196	0.4	42
24	294	165	0	0	207	0.45	32
25	277	156	0	0	216	0.5	24
26	532	0	0	53	175	0.3	91
27	493	0	0	49	190	0.35	75
28	460	0	0	46	202	0.4	59
29	431	0	0	43	213	0.45	45
30	406	0	0	40	223	0.5	40
31	473	0	0	104	173	0.3	82
32	439	0	0	96	188	0.35	66
33	410	0	0	90	200	0.4	51
34	384	0	0	84	211	0.45	42
35	362	0	0	79	221	0.5	35
36	416	0	0	154	171	0.3	70
37	387	0	0	143	185	0.35	62
38	361	0	0	134	198	0.4	49
39	339	0	0	125	209	0.45	37
40	319	0	0	118	219	0.5	32
41	360	0	0	203	169	0.3	59
42	335	0	0	188	183	0.35	47
43	313	0	0	176	196	0.4	39
44	294	0	0	165	207	0.45	32
45	277	0	0	156	216	0.5	28
46	532	0	53	0	175	0.3	83
47	493	0	49	0	190	0.35	76
48	460	0	46	0	202	0.4	57
49	431	0	43	0	213	0.45	52
50	406	0	40	0	223	0.5	46
51	473	0	104	0	173	0.3	84
52	439	0	96	0	188	0.35	62
53	410	0	90	0	200	0.4	53
54	384	0	84	0	211	0.45	43
55	362	0	79	0	221	0.5	39
56	416	0	154	0	171	0.3	73
57	387	0	143	0	185	0.35	62
58	361	0	134	0	198	0.4	51
59	339	0	125	0	209	0.45	43
60	319	0	118	0	219	0.5	38
61	360	0	203	0	169	0.3	66
62	335	0	188	0	183	0.35	57
63	313	0	176	0	196	0.4	48
64	294	0	165	0	207	0.45	39
65	277	0	156	0	216	0.5	34

Mix IDs 1–5: control group (pure cement paste). Mix IDs 6–25: limestone powder (ST) replacement. Mix IDs 26–45: fly ash (FA) replacement. Mix IDs 46–65: slag (SL) replacement. Each group includes mixes with w/b = 0.30 to 0.50, and the compressive strength results range from 24 MPa to 91 MPa, depending on composition.

## Data Availability

The original contributions presented in this study are included in the article. Further inquiries can be directed to the corresponding authors.
